# Contributions to Morbid Anatomy

**Published:** 1843-10

**Authors:** Bennet Dowler

**Affiliations:** New Orleans


### WESTERN JOURNAL

OF

### MEDICINE AND SURGERY

---

OCTOBER, 1843.

---

ART. I.—*Contributions to Morbid Anatomy.* By BENNET DOWLER, M.D., of New Orleans.

Chemistry offers one of the best illustrations of the great importance of a scientific NOMENCLATURE; a nomenclature full of meaning, easily learned because based on analogy, and sent forth to the world along with each new chemical product, and this too, in most instances, in advance of popular, unmeaning names. When a name is once fixed, especially if it be a short one, in vain may the learned labor to substitute for it a Greek, Latin, or even another English name, however absurd may be the former—however descriptive may be the latter. "All nomenclature," says Mr. Herschel, "must be a balance of difficulties, and a good short *unmeaning*

name, which has once obtained a footing in usage, is preferable to almost any other." (*Nat. Philos.*)

*Melan-orrhœa* is (if I might be allowed to coin a word) better than the inelegant and contradictory phrase, *purging* black *vomit; melan-emesis,* less tautological than *vomiting* black *vomit; melan-orrhagia,* less circumlocutory than discharges of blood and black vomit. Take another example: in yellow-fever, in the early stage, there is a pain in the frontal bone, above the eyes, never in them even when exposed to the strongest light, sometimes extending to the temples, but seldom, if ever, diffused through the head. This, though erroneously called head-ache by careless patients, and writers, is not strictly such. Now this pain is peculiar, not from its intensity, but from its location, a single finger placed above the eye-brows usually covering its seat, and it might be expressed, without circumlocution, by the term *frontalgia;* pain along the entire spine, might be called *spinalgia;* pain in its cervical portion, would by the same rule be named *cervalgia;* in the middle portion *dorsalgia;* in the lower, *lumbalgia;* more especially as *neuralgia* and several other words of this family are becoming anglicised. The shortest description for *lumbalgia* would, perhaps, in plain English, be this: pain in the small of the back, directly in the bone. However convenient such names may be, the history of medical nomenclature shows that they will, at last, be found to give place to a simpler and more vernacular technology, even among professors of the healing art. In many cases this tendency is advantageous. Who would wish to change the English and French names of yellow-fever, for any of those learned terms which Greek and Latin scholars have invented? The former name implies no false theories of malignancy, of putridity, of typhus, &c., but originates in a fact of constant occurrence in fatal cases, namely, a yellowness of the skin, of the subconjunctival, the adipose, and cellular tissues. The Spanish name, *vômito priéto,* is less characteristic, inasmuch as black-vomit is far from being constant in this fever, though even this name is, perhaps, more correct than the classical:

for black-vomit matter is generally found upon dissection, either in the stomach, or intestines, though in many, perhaps a majority of cases, none had been vomited.

The elaborate and classical nomenclature of the late, and very learned, Dr. Mason Good, after blazing comet-like in the medical heavens, is fast disappearing in the deeps of oblivion, a work which he evidently thought contained the elements of its own immortality—a work, as he declares, "not merely designed as a manual for the student, or a text-book for the lecturer, but a book that might stand on the same shelf with our most popular systems of Natural History." (*Prelim. Dis.* p. vii.) The same quotation, in which he bewails the short-lived glories of Darwin's Zoonomia, which, according to its author, was destined to "stand like a rock amid the waste of ages," might now be applied equally to his own case:

> "O blindness to the future!—kindly given,—

with the following additional lines from the same poet:

> "Ev'n he whose soul now melts in mournful lays,
> Shall shortly want the generous tear he pays:"

since Dr. Good's Nosology too, "must fall like those he sung."

I trust that I shall not be accused of a desire to innovate, in offering to the consideration of dissectors the following plain names, with some account of the anatomical characters which they represent, without entering for the present, the more elevated range of pathological inquiry to which they might have a tendency. I am not aware that all these lesions have been designated and introduced into works on anatomy, though I believe they are entitled to such distinction, or at least to a hearing. It is not my intention to discuss their practical value, still less to attempt by deductive reasoning to trace their consequences and bearings through the extended realms of pathology.

I do not insist upon the adoption of the following names, nor venture, in all instances, to use them in my own descriptions, however convenient they might be, in order to avoid tedious circumlocutions. It is hoped others will substitute names better, shorter, and more descriptive of the *things* designated. It requires a celebrated name to establish new names. Andral is an instance: a part of his pathological nomenclature spread with celerity from the centre to the circumference of Europe, as well as to the furthest verge of America.

I.—Cellular Hyperæmia, or Cellular Spleenification of the Extremities.—*Ante-mortem History:*—The skin natural in color, but tense; the depressions among the muscles obliterated; the limb rounded; extension, as well as any kind of motion, more or less painful, semi-flexion affording relief; the limb is but little increased in size: these symptoms seldom causing much complaint.

*Anatomical Characters:*—The interstices of the cellular membrane uniformly infiltrated with a semi-carnified blood, firmer than the spleen, a little variegated in some instances with gray or buff color, presenting when cut, a smooth surface, free from serosity and bloody exudations; sometimes affecting only a portion of the limb, sometimes the whole of it, sometimes all the limbs, though never any portion of the trunk so far as I have seen.

The following extracts, taken from my manuscript chapters on the morbid anatomy of yellow-fever, will illustrate this lesion: J. M., born in New York, musical instrument maker, aged twenty-three, on the third day after his arrival at New Orleans, took the yellow-fever. Body of medium size, muscular and adipose tissues finely proportioned, &c. [His senses and his susceptibility to external impressions continued good until fifteen minutes before he died. Daily history omitted.]

Oct. 7th, 1842.—Has no fixed pain; but can, at any time, produce pains in his limbs by certain motions, as extension and elevation. The limbs, which are rounded and somewhat tense, he keeps semi-flexed.

Oct. 12th, at 11½ A. M., after a slight effort to vomit for the first time, he complained that his feet were becoming cold, at the same time expressing an opinion that he would recover, and in a few seconds after expired tranquilly. The day before his death, the uneasy sensation in his limbs existed, as at first, whenever he moved them.

Dissection fifteen minutes after death:

*Body*, warm, except the feet; muscles contractile, firm, well developed, and slightly increased in redness, &c. The cellular tissue, even in the deepest recesses of the muscles of the arms and legs, was loaded with semi-organised blood, which, when cut, resembled smooth, dense jelly, interwoven with a tissue like spleen or placenta—free from serosity or bloody oozings. This deposit occupied more than half of the cellular tissue, excepting the right arm, which was less affected.

There was no hæmorrhagic appearance in the bowels or in any other organs. The blood was natural in color, and in consistence, coagulating firmly as usual.

There can be no doubt that this lesion occurred early in his malady, certainly five days before death. Not wishing to encumber this paper with histories of a similar character, I proceed to another lesion, which, though it is identical in color, differs in its location, shape, and consistence.

II.—Button-shaped Sanguineous Indurations.—*Anatomical Characters:*—These bodies, less organised than the spleen, firmer and darker than coagulated blood, resemble dense jelly in consistence, occupying and being intersected by the submucous tissue of the stomach and intestines—of the urinary and gall bladders, varying from minute points to the size of a common button, which they resemble in shape. Conceive the eye or thick part of a button as commencing in the mucous membrane, and the body as extending back through the submucous tissue, the muscular coat moulding the outside, and you will have a correct idea of their general configuration. This lesion happens oftenest in the stomach, and is not uncommon, at least, in yellow-fever. In its solidity and well-defined shape, it differs greatly from those passive exudations which take place in the last stage of fever, when the injecting power of the circulation must be too feeble to force the blood and compress it into the tissues as above described. This lesion has been found in subjects even where blood-letting had been carried to a state bordering on *anæmia.*

In the descending scale, as connected with this subject, I think it by no means ridiculous or unimportant to mention certain appearances, if not strictly lesions, of the skin in the living, and in the dead subject, commonly called *petechiæ*, but which generally are, in my opinion, neither more nor less than

III.—Mosquitoe Petechiæ or Ecchymoses.—These are found only on such parts as are exposed to these insects. They are of no small value as indicants of a hæmorrhagic diathesis or tendency. The same may be said of that dark, purplish-red discoloraton which is apt to appear to considerable extent around the neatest orifice in the arm in venesection, though in neither case do we meet with sanguineous concretions as in the last lesion.

Mosquitoe Petechiæ I have met with in several intense forms of remittent and congestive fevers, during the present month (July 13th, 1843); every exposed part being thickly dotted with circumscribed, red spots, seldom as large as a grain of flaxseed, the color receding by pressure as in erysipelas, but returning almost instantly after the removal of the finger. These dottings occur sometimes in bowel affections, as diarrhœa, dysentery, and cholera morbus. In healthy infants, recently born, the mosquitoe bite causes spots difficult to be distinguished from those above described. Pringle, (*Dis. of the Army*), says that *petechiæ* appear thickest upon the breast and the back, less on the legs and arms; and, adds he, "I do not remember to have observed any upon the face." (Obs. on Jail or Hospital Fever, p. 262). In mosquito petechiæ, the contrary happens. If the patient's shirt be of very thick linen (as in the Charity Hospital of this city) no spots will be found on the trunk, except at the opening in the bosom, unless the body be exposed, as in delirium.

De Haen, nearly a century since, characterised typhus as a petechial fever (*frebris petechialis*), and it is now considered by Louis and Chomel, as possessing no other pathognomonic character. Since pathologists attach so much importance to petechial eruption, it is desirable to discriminate its varieties as accurately as possible. It will be found, in the South at least, as I incline to believe, that those spots called *petechiæ* are owing to the bites of insects, in almost all instances. The advanced stage of fever, the prostration of the vital forces, and a hæmorrhagic tendency, are circumstances generally connected with this appearance. These bites, however, seem to produce no uneasy sensations as in healthy persons: upon the latter, the mosquitoes sometimes produce irritating effects not a little formidable, through nearly half the year, in this city; though they are far less virulent than Capt. Ross represents them to be in the arctic regions, at the 67° N. lat. (2d Voy., 1829, p. 35).

Lastly, upon this branch of our subject, is there nothing morbid in the firmness, tension, plumpness, and redness of the muscles so common in yellow-fever subjects? Would not an apoplexy, or engorgement of the interstitial and capillary circulations, or either of them, appear probable under such circumstances? Blood-letting may be carried to great extremes, causing general *anæmia*, without discharging this fine red color of the muscular tissue.

IV.—Cord-like Consolidation of the Intestine.—*Anatomical Characters:*—The large intestine chiefly, though not exclusively, is the seat of this lesion; the cæcum, colon, and rectum, are found firm, round, bloodless—strong, tenacious, elastic, white—often resembling in color the breast of a boiled chicken, or prepared tripe; the intestinal cavity obliterated, not admitting a body as large as a quill without forcible dilatation; dessication of the mucous membrane; all the coats much but equally thickened; the external dilatations, notchings, and puckerings, as well as the internal sacculated pouches obliterated, leaving the longitudinal muscular stripes distinct; the consolidated portion probably diminished in length; in most cases empty; sometimes containing solid pebble-shaped fæces, from the size of a hazel-nut to a hen's egg, or even to a goose's egg. These indurations, when numerous, give the large intestine the appearance of a string of onions, being of the consistence and color of half-dried or baked yellow clay, closely surrounded as with a skin, by the intestine, emitting little if any odor.

If we deduct for the apparent loss in the mucous, serous, and sanguineous fluids natural to the parts, it is probable that the bowel has suffered no loss of weight by this lesion. This change, which is a very acute one, since it happens for the most part in fevers lasting from three to four days, is quite different from atrophy, or collapse, which happens to a muscle or organ from long disuse of its natural function. It does not appear to be occasioned by mere emptiness, a condition to which the large intestine is often subject in sickness and health—distention appearing to be its natural condition—differing in this respect from the urinary bladder, which habitually contracts or dilates more or less, according to the quantity of fluid which it may contain: on the contrary, the large intestine when most empty of fæcal accumulations, is most distended with its peculiar gases, at least, such is often the case in the dead body.

There can be no doubt when this lesion is established from the valve of the cæcum to the anus, as is often the case, that defecation cannot be effected; and, therefore, when purgation is essential to life, this lesion must be highly dangerous or absolutely fatal. From the universal stricture, dryness, and, sometimes, almost cartilaginous firmness of the intestinal walls, enemata, or gum-elastic flexible tubes would alike fail in many cases to render the canal pervious for the purposes of defecation. When we reflect on the anatomy of the colon, including that enormous triangular bag called the cæcum at its origin, its great size, its constant gaseous distention, its thinness (being naturally the most attenuated of all the bowels), it appears difficult to account for this lesion. Do the muscular fibres of the intestines undergo a rigidity and contraction, analagous to tetanus or lock-jaw in the external muscles? Is this an intestinal tetanus, so intense as to survive the relaxing power of death itself? From the thickness, muscularity, and diminished calibre of the rectum, one might expect that this portion of the great intestine would be more completely closed than any other; but, the contrary happens. Two or three inches above the anus, small portions of enemata are sometimes found.

Without entering upon the pathology of this lesion, I may be allowed to remark, that I have heard a number of yellow-fever patients affirm that, during their whole illness, they never had a single defecation or stool. I have known several, both in convalescent and fatal cases, who, after free purgations during the first day, had no further defecations, or such, only, as I have often found in this lesion, and usually described by the patient as small dry pills. Some of these patients had taken quinine. Is not this salt a constringer of the bowels? I regret that my attention has not been arrested by this inquiry in time to afford me a sufficient number of observations upon which to form a satisfactory opinion. The advocates of the quinine practice would do well to inform the medical public on this subject, as nearly all other modes of treatment, in which quinine is only an occasional element, are too complex to afford exact and unmodified results.

Though this lesion is not positively known to have any connexion with the quinine practice, yet, such practice may increase the morbid alteration, instead of diminishing it.

The above description, it is believed, will apply to a majority of cases in which this lesion is found, though it will, as might be expected, transcend the appearances in some instances: with these exceptions, the picture I have drawn, will not, I trust, be chargeable with exaggeration.

The ante-mortem history or symptoms of this change, though little known, will doubtless be ascertained to a greater or less extent before long. A timely and well conducted course of purgation, with enemata or lavements, assisted with suitable instruments, would probably prevent this affection altogether. Enemata though greatly and generally overrated by physicians, by patients, and by the public, as I humbly conceive, might be expected to act beneficially in preventing or lessening this lesion.

*Case.*—Large man from Louisville—the cæcum, colon, and rectum contracted to the size of the thumb, scarcely pervious to the scissors, containing nothing but a handful of small, dry, inodorous, friable, black crumbs or scybala, from the size of a pea to a hazel-nut; the whole of the large intestine being firm, strong, elastic, thickened, blanched, bloodless, and scarcely moist within. I add the following facts for comparison: mouth and gullet full of blood; stomach small, blanched, four ounces of black-vomit, without any bloody tinge; jejunum, blood and black-vomit; the upper third of the ileum gray chylous paste, the residue black-vomit again.

V.—Diminished adhesion of the Hepatic Coats.—*Anatomical Characters:*—The coats bloodless, and non-vascular; on making an incision both the cellular and serous envelopes peel off with facility, in flakes as large as the hand, like the skin from a boiled potatoe, scarcely ever bringing any portion of the parenchyma of the liver; sometimes, the serous coat alone is non-adherent, separating from the cellular coat, which latter maintains its natural adhesions; these membranes appear nearly transparent, very thin, without loss of tenacity, free from blood-vessels, diminished in moisture; the substance of the liver appearing, when denuded, either soft, dry, and brittle, or firm, dry, and brittle, and nearly destitute of blood. If we imagine a liver half-boiled, then macerated, and afterwards partially dessicated, so as to weaken its membranous adhesions, at the same time preserving their strength, and discharging their blood—we will probably have an apt illustration of this lesion, a lesion by no means infrequent or unimportant in the pathology of the liver.

May not the life of the hepatic coats cease, a considerable period before the death of the general system, thereby causing diminished adhesion, loss of moisture, non-vascularity, and anæmia?

VI.—Oyster-like degeneration of the Gall-bladder.—For this comparison I am indebted to a suggestion made by

Dr. C. A. Luzenberg, of this city. This lesion is very common, at least in yellow-fever. The gall-bladder is usually whiter, firmer, tenser, tougher, and larger than an oyster of common size; its walls, from a quarter to half an inch thick, consisting of innumerable threads or fibres, like raw cotton, intersecting a dull-white, semi-opaque, gelatinoid, semi-organized substance, having but little or no serosity; the cavity of the gall-bladder, usually containing a thick and apparently pure albuminous substance, without a trace of bile, though in some few cases, grass-green and dark tenacious bile, and biliary concretions from the consistence of soft sugar, to hard aloes, exist simultaneously with this lesion; the serous, cellular, and mucous coats, free from vascularity, as well as the adjacent parts of the liver; in a few cases button-shaped, though minute, sanguineous extravasations are found under the serous coat—more seldom a faint, bloody tinge in the deposits of the cellular coat.

This lesion does not resemble œdematous infiltration, and is unaccompanied with anasarca in any part of the body. Its firmness of texture and its destitution of serosity show that it is not a dropsy. The hypertrophy of the walls of the gall-bladder, is often attended with a contraction of its cavity; the latter sometimes contains from one to two ounces of albumenoid matter, nearly as transparent as water.

The following instance of this lesion, is taken from a robust subject, from Louisville: it is not so fully described as many others; and the biliary concretions must be regarded as infrequent, though sometimes met with in the anatomy of this malady: The gall-bladder enlarged, walls thickened to half an inch, densely infiltrated with a solid albuminous or gelatinoid substance, giving the whole a dull-white oyster-like appearance; its cavity contained about one ounce of liquid exactly like the white of an egg, free from bile; also, twenty irregular stones, of a dark color, sinking in water, crumbling on firm pressure like fine aloes, being brittle, not staining the hand. On exposure to the air their brittleness increased, so that in falling they broke with a smooth fracture; insoluble in water; some weighed eight grains each. [Now, after nearly two years they are gray and firm, and have an astringent metallic taste.] These concretions had no nuclei or strata. The mucous coat of the gall-bladder, natural, except its white color.

The interstitial deposite resembles fibrinous or lymphy concretions, rather than serosity. In color, not in consistence, this lesion resembles *œdema of the glottis*, which may be referred to for the purpose of illustration. The following case I saw through the politeness of Dr. Wederstrandt: A young man who died, suffocated, September 11th, 1842, presented the following appearances upon dissection, the same day: The posterior fauces, the glottis, epiglottis, and larynx, presented a uniform white, non-vascular tumefaction, free from ulceration; the sub-mucous tissue in some places, half an inch thick from serous infiltration, being semi-transparent, free from redness, the lips of the glottis touching each other, closing the air passage; the substance of the lungs infiltrated with serosity and with gas.

In the above described change of the gall-bladder, the serous infiltration, the spongy, and the doughy texture of anasarca, are absent. The coats and ducts of the gall-bladder, together with the neighboring portions of the liver and its coats, are free from vascularity, redness, congestion, and swelling. The condition of the liver, usually accompanying this lesion, so far from favoring the idea of serous infiltration, presents more or less dessication, with, in many cases, diminished cohesion of its parenchyma. Whether it be an inflammatory or a non-inflammatory change, I will not undertake to say. If regarded as the former, it affords another instance of the difficulty of defining inflammation, seeing redness, vascularity, &c., are wanting.

May not the absence of the accustomed bile, prove a source of irritation to the gall-bladder, thereby laying the foundation of this alteration. Though, this may be only a sub-lesion, a wheel in a wheel, yet, as it sometimes nearly obliterates the cavity of the bladder and its ducts, it might possibly by mechanical obstruction do infinite harm.

VII.–Yellow, brittle dessication of the Liver.–*Anatomical Characters:*–Dull-yellow, cork, beeswax, or gingerbread color; parenchyma brittle and bloodless, and softened, with more or less dryness, especially in its thicker and more central portion, as if the liver had been boiled,—its parenchyma deprived of blood, and suppleness; breaking somewhat like shortened bread, though appearing destitute of any oily matter; sometimes moderately indurated, but differing from the hard, granulated, hob-nail, or cirrhose liver; never being accompanied, as the latter almost constantly is, with dropsy of the abdominal cavity.

This lesion, whether it belong to inflammatory or non-inflammatory changes, or be the effect of paralysis in the vessels concerned in the capillary circulation, must be regarded as very important in its pathological bearings.

The two most remarkable circumstances in the ante-mortem history of this lesion, seem to be, first, the absence of bile in the fæcal evacuations; and, secondly, tenderness in the liver when pressed in any direction. In order to avoid the cardia, pylorus, duodenum, gall-bladder, great nerves, and their ganglia and plexuses, and the pancreas resting on or near the spine, we may, after relaxing the abdominal muscles, direct the pressure upward against the diaphragm or upwards and outwards against the ribs and right side; or place the fingers on each side of the liver pressing them together without forcing it against the spine. With respect to bile, let it be borne in mind, that when the patient is attacked with fever, his bowels are sometimes loaded with healthy fæces, which accumulate in the large intestines, and there remain without much alteration until death, though in the mean time digestion and the secretion of both healthy and morbid bile, may have totally ceased. There seems to be an increase of heat in the integument, radiating from the liver. These, though the best diagnostics of this lesion that I know of, are nevertheless very unsatisfactory, and therefore, in the absence of better proof, I may be forgiven for drawing a little from what, perhaps, may be considered imagination. May not this lesion result from fever in the hepatic system (analogous to idiopathic fever in the general system); attended with a kind of semi-combustion, a condition somewhat like approaching spontaneous or idiopathic gangrene, resembling in cohesion or brittleness vegetable matter, such as masses of hay, straw, &c., after being heated to a degree short of perfect combustion.

In this lesion the large vessels are generally filled with blood, perhaps congested so far as the vena portæ and its principal branches are concerned, while the substance of the liver appears bloodless, indicating a paralysis or failure in the capillary and interstitial circulations to a great extent, and referable to an era in the malady considerably anterior to death.

Without adopting the conclusions of the ingenious Liebig, who seems to put pathology, physiology, therapeutics, and the animal kingdom, into his crucible, in order to furnish under the imposing name of animal chemistry, a panacea for the uncertainty of medicine, we may remark that one of the properties which he ascribes to the bile, is that of keeping up the animal heat, by a sort of combustion, "by presenting carbon and hydrogen in a soluble form to the oxygen of arterial blood;" that from seventeen to twenty-four ounces of this "vehicle of carbon and hydrogen," this fuel called bile, are secreted daily into the bowels to be taken up into the circulation, disappearing in the form of carbonic acid and water: now as the bile ceases to be secreted from the liver, the Liebigian theory would seem to suggest that this enormous amount of concentrated fuel, after neutralising the oxygen present in the liver, might like a smouldering fire carbonise the organ in which it originated or was retained, thus committing a sort of pathological suicide, by preying upon itself.

If we view the liver as the great organ of depuration of the general system as well as of the hepatic system, it may, like the poisonous snake which bites itself, die from its own poison—from a morbid retention of its own secretions, when suddenly arrested—functional causing organic alteration.

In this lesion does not the liver die in advance of the general system? May not its brittleness, its paleness, and the diminished adhesion of its coverings, have some connexion with such a cause?

VIII.—Serous compression or congestion of the Brain.—Considering the mass of names, and especially of synonymes, under which our science groans, it is desirable not to add any more to the ponderous load, without good reason. The above named lesion cannot, however, be ranked under that protracted disease called hydrocephalus, nor with that sudden affection, serous apoplexy, much less with the water-stroke of the Germans; nor is it identical with sanguineous apoplexy, sanguineous congestion, cerebritis, meningitis, arachnitis, &c. Whether it be a primary, or secondary lesion, I shall not stop to inquire. It is often complicated.

I do not propose to enter upon the ante-mortem history of this lesion, such as erysipeloid flushings of the face, throbbing and enlargement of the temporal arteries, pain across the forehead, vertigo on rising, inability in most instances to tell a continuous or circumstantial story, disposition to slumber, ending often in coma, seldom in furious delirium; occurring mostly in fevers, sometimes in consumption, dysentery, &c., in the last stages.

In this lesion the substance of the brain appears natural, and its envelopes are in some cases but little changed; the ventricles contain more or less water; the principal part of the serosity, varying from two to eight ounces, is found in the sack formed by the arachnoid and its reflexion upon the inner surface of the dura-mater, in other words, on the outer surface of the cerebral arachnoid, constituting a stratum of water around each hemisphere, and, of course, compressing the brain in all cases.

IX.—Vascular Collapse.—Opposed to vascular turgescence or injection: the *character* of this lesion is sufficiently explained by its name; the vessels are numerous, enlarged, perhaps varicose, but empty, and collapsed. This appearance is often found in the envelopes of the brain, in connexion with serosity; it may be partly owing to the manner of conducting the dissection, to cadaveric hyperæmia, and other causes, but in any case, it is of much importance, indicating previous hyperæmia, congestion, &c.

X.—Icteroid Deposite.—*Anatomical Characters:*—Cutaneous, adipose, cellular, and sub-conjunctival tissues more or less yellow—in many instances, with sub-arachnoid, and sub-peritoneal gelatinoid exudations, of a yellow color, especially along the course of the lymphatics and their ganglions, near the spine; between the posterior doubling of the mesentery, and in the anterior doublings of the mediastinum under the sternum; with yellowness of the liquor pericardii, but without serosity in the cavities, or in the cellular membrane.

The inquiries into hepatic pathology, suggested by this lesion, are too extensive for the present occasion. *Icteroid deposite* is evidently identical with, or occasioned by bile or some of its modifications. This appearance of the skin and fatty tissue can be produced at pleasure, in the healthy subject, by spreading diluted bile over the skin, and other white tissues.

The Boerhaavian notion of an *error loci*—a doctrine which supposes the circulation of the blood, lymph, and serum, to be carried on by vessels of different calibres, and that the large globules are sometimes forced into the small vessels, occasioning obstruction by an *error of place*—how erroneous soever it may be in its application to the general circulation, has nevertheless one feature (*error of place*) which seems to apply to this lesion in the biliary circulation. This can scarcely be called theory: though, on tasting the icteroid tissues, I have not been sensible of any decided bitterness, yet the color is too exact to admit of any mistake.

The yellowness of the eyes, always attributed, though erroneously, to the sclerotic coat, is confined to a dense cellular web between it and the conjunctiva. This web I accidentally noticed, as early as 1841, and, was not aware, until the present year (1843), that Dr. Gross had first presented a description of it, in 1839, in nearly the same words which I generally used: he calls it "the *ocular fascia*—a pretty thick layer of cellular tissue," &c.

The following is offered as an example of *icteroid deposite:* A large, middle-aged man, from Louisville, on the 4th of November, 1841, (then five days sick), walked up several long stairways, throwing up black-vomit; senses regular; died fourteen hours after, having used only cooling drinks, &c.

Dissection ten minutes after death:—Skin and sub-conjunctival tissue, yellow. The weight of the body estimated beyond two hundred pounds. Adipose tissue on the chest, abdomen, and thighs, one to two inches thick—the omenta and the mesentery, about the same; the large intestine almost completely enclosed in a cylinder of fat, nearly an inch thick; the kidneys and mesenteric glands, concealed in masses of fat. The fat in the interior estimated at fifteen pounds; that of the exterior three or four times greater; the whole being firm, free from serosity, but every where of a pale, golden color.

The following is a remarkable case of icteroid deposite in all the tissues, susceptible of reflecting a yellow color, the bones excepted. It is proper for me to inform the reader, that while I regard this as a case of yellow-fever, the symptoms did not seem fully to warrant that opinion, until the last or dying stage, which, in connexion with the dissection, appeared decisive. Some very respectable medical gentlemen regarded this as a case of jaundice; others, not less competent, as a case of yellow fever. It matters but little whether the name be jaundice, ague, or parturition, if facts be given.

July 11th 1843.—L. F., born in France, aged twenty-seven, last from Havana, wood-cutter, resident three years, admitted yesterday into the Hospital, sick one week; speaks French only; medium in size; adipose tissue scanty: rests on his back; has a little pain in his forehead; quiet; senses natural; countenance free from stupidity and dejection; respiration, abdomen, pulse, tongue, and temperature of the skin natural; liver, slightly tender upon pressure; skin of an intense yellow, inclining to dusky hue; eyes, yellow without injection. Cups—Nit. mur. acid bath.

July 12th.—Pulse, breathing, tongue, and epigastrium, natural; yellowness undiminished. Sponging; 5 grains bluemass; gum-water.

July 14th.—Senses natural; restless; copious hæmorrhage from the mouth and nose; throws up much black-vomit.

July 15th.—Black-vomit. Death, 10, A. M.

Dissection two hours after death:

*Body* moderately emaciated, warm; muscles natural; blood, dark, coagulating firmly. In an hour after the removal of the lungs, a clot formed in the pleural cavity along the spine, about a foot and a half in length, and nearly two inches thick, so tenacious and solid that it was lifted out of the chest entire, and, being suspended across the subject's leg for two hours, remained unbroken, having a dull grayish and yellowish color upon its surface. The liver, natural in size and consistence, had a slight mixture of dull gray and cork colors; gall-bladder, distended with a dark colored, rather thick bile, flowing freely through the common duct into the duodenum, by moderate pressure. Fluid and flaky black-vomit in the stomach; blood and black-vomit in the bowels. The skin, eyes, omenta, mesentery, pancreas, portions of the kidnies, urinary bladder, tendons, fasciæ, periosteum, the adipose, serous, mucous, and fibrous membranes in the head, chest, and abdomen, more or less yellow, often of a golden hue, especially in the great tendons, cartilages, and fasciæ.

XI.—Black-vomit Petechiæ.—*Anatomical Characters:*—Small dottings in the mucous tissue of the duodenum, stomach, and small intestines, sometimes like grains of powder, or Indian ink, with which sailors and others mark their bodies with names, pictures, &c., but more generally, like fine, black sand, or charcoal powder; always black; not removable by washing, though not deeply imbedded in the tissue, which is generally of a natural consistence.

I take the following case, at random, from the dissection of a stout man from Louisville: The mucous coat of the duodenum thin, rough or mammillated, and infiltrated with a fine dust or a powder-like substance, black as charcoal, not removable by washing or gentle friction, but imbedded in the tissue. These small specks, resembling those sometimes seen on the surface of apples, were distinct, but very numerous, giving the membrane a black or gray appearance, according to their concentrated or isolated location. No change of texture in the other duodenal coats.

Is not this the black-vomit matter *in transitu?*

XII.—Œsophageal Apthæ.—*Anatomical Characters:*—Irregular, generally longitudinal patches, and stripes in the epidermic lining of the gullet, without marked change in the mucous tissue, which latter suffers more or less denudation; the white portions of the epithelium, brittle, easily detached. The state of the mouth, stomach, intestines, and gullet itself, do not appear to offer any uniform accompanying appearances illustrative or explanatory of this lesion, which much resembles, at least in color, the mildest forms of thrush or apthæ in children, and is, probably, of no great importance in morbid anatomy.

XIII.—Solar Hyperæmia of the Lungs.—The history and morbid anatomy of this lesion are given in two papers which appeared in the New York Medical Gazette, in the year 1841, one of which was copied into this Journal. (Vid. vol. iv, p. 384, November, 1841—*Observ. on Solar Asphyxia or Coup de Soleil.*) The subject is now alluded to chiefly for the purpose of suggesting a name for the lesion which is there fully described, as well as its ante-mortem history.

This lesion, nearly perfect, has been found in a few instances after rapid deaths from yellow-fever: a man from Louisville, died of yellow-fever; I was informed that he had, in the *last* stage, symptoms resembling *asphyxia.* Dissection ten minutes after death: the entire mucous membrane of the air-passages of a dull red color, loaded with blood; lungs completely distended, filling the chest and crepitant only upon their exterior portions; extensive central infiltration with solidified, jelly-like blood; the pulmonary tissue on being cut in slices presented smooth surfaces, impermeable to air; the bronchial tubes contained bloody froth. This dead subject has been referred to more than once, in this paper, for illustrations.

I hope I may be permitted to add, not as lesions, but as the names of facts not unworthy of the dissector's attention, the following:

XIV.—Post-mortem Muscular Contractility.

XV.—Post-mortem Capillary Circulation.—In the April number of this Journal, (Art. *Post-mortem Researches,*) these facts are shown to have an application to the physiology and pathology of death. Let it not be supposed that these terms are absurd. They relate to that middle state between apparent and absolute death—that *terra incognita*, where vitality, though extinguished in some tissues, smoulders in others, or quickly disappearing from all, is succeeded by the reign of decomposition, obscuring the foot-steps of disease, like a volcano whose eruptions having subsided, leave the air darkened with cinders and ashes, which, continuing to fall, not only obliterate the traveller's pathway, but conceal the rents or lesions which have, on all sides, scarred and disfigured the face of nature.

Death—The Agony.—The transition through the territory of death—the change from vital to inert matter, presents a sort of interregnum in physiology, and in pathological anatomy, of which imagination has taken advantage, by exaggerating the pains and horrors to which it is incident. Even the language of science is tinged with unwonted severity. The last stage, when, if the patient's understanding be unimpaired, he seldom complains except of inability to sleep, loss of appetite, want of exercise, slight smothering, &c.—the last stage, when often, a deep sleep, with total insensibility and unconsciousness, exclude the possibility of acute suffering, is called the agony—a phrase by far less applicable to death from acute fever, especially and beyond all others yellow-fever, than to deaths from consumption, dysentery, and some other maladies. In the agony, I have known men to talk about small uncollected debts, and matters of far less importance, or of total indifference, expressing no painful sensations when asked in relation to the subject. I have seen them watch the mosquito to brush it off, and wipe with care black-vomit from their bed-clothes. Particularly have I been struck with the ingenuity with which the mothers of families have interrogated me in the LAST AGONY as to the chances of their recovery, when in fact they felt nothing scarcely of death's advances at the time.

In the last agony the poet Keats said, "I feel the flowers growing over me." The last words spoken by the astronomer Laplace, were: "*Ce que nous connaissons est peu de chose; ce que nous ignorons est immense.*" In the last agony, Dr. Wm. Hunter said to his friend Combe: "If I had strength enough to hold a pen, I would write how easy and pleasant a thing it is to die." Dr. Geo. Fordyce, in the last agony, "desired his youngest daughter to read to him. She read for about twenty minutes, when the doctor said, 'stop, go out of the room, I am going to die'—the attendant went immediately into the bed-room and found him dead."

The following case which I clip from my MSS. outlines of the symptoms of yellow-fever, as illustrated by special histories taken at the bedside, will show that, from first to last, there is not in some cases of this malady any acute agony at all:

Bonar, born in Ireland, aged about thirty, a pedlar in Girod street, of strong constitution, resident in New Orleans seven months, requested my services, at four o'clock, on the evening of August 13th, 1839. He had walked about during the day without being aware of any fever, until the afternoon: understanding natural; *has no pain;* said he could "drink the ocean dry," and felt a smothering at his heart; pulse air-like, without being rapid; skin cool, dusky, yellowish, and moist; eyes red, prominent, with considerable yellowness; tongue reddish, dry, rough; nausea; breathing easy; bowels costive; has taken no medicine. Ordered blue-mass, calomel, sinapisms, bath, &c.; medicine operated mildly in the night; he slept a little twice. He had hiccup; a sensation of suffiocation; wished to go out into the open air. At 7 o'clock next morning, he sent a police officer to know if he might take a glass of wine or spirits. In five minutes after (before I had time to go two squares) he died; having lived about fourteen hours, from the time at which he first felt any serious symptoms. His wife, fair, ruddy, robust, and young, died with black-vomit, accompanied with abortion, five days before him, leaving a family of young children among strangers. I did not see her until the last stage. Her understanding was clear. Two hours before death, in the most forcible and artful manner, she endeavored to draw from me a direct opinion of the result of her case. She suffered but *little—no pain* except from pressure on the abdomen.

Conclusion.—In drawing up this brief account of the above lesions, I have often trusted to memory, though I could have copied a great many histories made when the subject was actually before me, a proceeding too extended for Journalism.

Since these observations, such as they are, did not originate in the closet, nor yet in a mere compilation of what others had done, I shall deserve the less censure, if in some instances memory has led me into any exaggerations—they are wholly inadvertent. It is possible that some terms used in these descriptions, though exact and just in their application to particular cases, may not apply equally to the average character of the lesion. All I can say is, that I have seen, or I believe I have seen in the dead body, what I have here related.

Written, as this paper is, in the hurry incidental to professional business, I have paid but little attention to its literary character. Without wishing to overrate or even to appreciate, at this moment, the importance which these lesions may possibly possess, without attempting to redeem their barrenness by pathological discussion, I consign them to the attention of the student of morbid anatomy. I regret that I have not leisure, perhaps not a sufficient number of observations, to ascertain by calculation their relative frequency.

Having no theoretical Eureka to uphold, like Adam Warner, in the Last of the Barons, I have not even attempted systematic arrangement in this paper. The Eclectic school, so catholic in its aims, choosing from all systems whatever is the best established as general principles, is the true medical church, which Hufeland has described: "there has ever been an invisible church of true physicians who have continued faithful to nature, have acted under her direction, who have all thought and meant the same thing, who will be understood throughout all ages and in spite of the confusion of tongues." To eclecticism, not to exclusivism, must we look for our morbid anatomy. "It would be absurd to ask the dove to write the natural history of the eagle; it *must* be one sided." (*Mrs. Austin*). The pendulum of theory now vibrates between solidism and humoralism. It may be said there is at this moment an undue bias towards the latter, though there is little or nothing new in the way of observation and experiment, to justify such predilection. As yet the facts offered are too much like the charge of a celebrated judge to the jury:

"Chief Justice Parker,
He made that darker,
Which was dark enough before."

Those who are now fully satisfied with former theories must be so upon the principle by which Ottilie explains resignation—"Resignation, my dear, is only despair."

Genius will ever theorise, and so it should, with proper restrictions and limitations. "All genius is metaphysical; because the ultimate end and aim of genius is ideal, however it may be actualized by incidental and accidental circumstances." (*Coleridge*). Even the rejection of all theory, is in itself a theory. Eclecticism consists of a series of theories or rather of general principles, more or less established. Exclusivism is what the Indians call a medicine-man; by its influence, rather than by its truth, it tyrannises over the minds of the multitude.

Pathological anatomy, alike useful in overthrowing false theories, and in laying the foundation of true principles, is now advancing with an unparalleled rapidity. "The force of intellect all-powerful in the review of the past, is seldom felt in judging of the present." (*Alison's Hist.*) Yet, here, the past is almost nothing—the present almost every thing. If we except a few illustrious names, recently numbered with the dead, what can the past offer in morbid anatomy, worthy of comparison with the works of Louis, Andral, Cruveilhier, not to mention other names now living, and destined to live longer than the pyramids of the Pharaohs.

In pathological anatomy, the range of legitimate theory is, as it ought to be, a limited one. It marshals facts into their proper places, agreeably to their family resemblances, analytically and synthetically. Its deductions are only applications of general facts. Its inductions, ascending from facts to facts according to their order of succession, discover more and more the uniformity and the universality of the facts themselves, without vainly seeking to show, what has never been explained, the absolute or essential connexions between cause and effect. Let those anatomists who explain every thing by the knife—let those pathologists who trace the causes of disease through their chemical processes, reflect upon the following explanation of cause and effect, by Dugald Stewart:

"It seems now to be pretty generally agreed among philosophers, that there is no instance in which we are able to perceive a necessary connexion between two successive events, or to comprehend in what manner the one proceeds from the other, as its cause. From experience, indeed, we learn, that there are many events, which are constantly conjoined, so that the one invariably follows the other; but it is possible, for any thing we know to the contrary, that this connection, though a constant one, as far as our observation has reached, may not be a necessary connexion; nay, it is possible that there may be no necessary connections among any of the phenomena we see: and, if there are any such connections existing, we may rest assured that we shall never be able to discover them." (*Elem. Philos. Mind.*)

How grating to the ears of the men who have been accustomed to hear the above language from the lips of the late Professor Stewart, of the University of Edinburgh, must be the following language of Professor Liebig, who is expected to succeed to a chair in the same University, during the present year. After asserting that, "quinine, the alkaloids of opium, morphia, &c., combine chemically, by means of their elements forming new, or transforming existing brain and nervous matter—become converted into their constituents"—(how much better to give the genuine article, *brain itself!*) Professor L. holds the following language: "Their action on the healthy, as well as diseased organism, admits of a surprisingly simple explanation. It argues only a want of sense to consider the action of these substances inexplicable." (*Animal Chemistry, appli. to Phys. and Path.*)

A fact, in popular language may be said to be explained, when it is referred to what appears as its immediate cause, though this, in truth, is only saying that one thing succeeds another, and is, strictly, no explanation at all. Pathological explanation, in a strict sense, is too deep to be fathomed with the longest sounding line of sciolism, or of *animal chemistry itself*.

Destiny, was to Jupiter, gods and men, what theory is to physicians; it directs the pen from the beginning to the end of every prescription. When the star of Broussais was at its culmination, in 1833, France, once a large exporter of leeches to the number of millions, exhausted England, Germany, Hungary, Moldavia and Wallachia, importing in a single year, 41,654,300! (Vid. *Dr. Muehry's State of Med*). A thousand scalpels gleamed almost at once in the realms of the dead—"La France," said Cruveilhier, "est désormais la terre classique de l'anatomie pathologique." (*Anat. Path.* p. 5). Nor was this a vain boast, undeserved by the *grande nation*. Morbid anatomy soon clipped the wings of Broussais, diminished *gastrites*, and lessened the importation of leeches; and, long before this great man died, his doctrine, his popularity, and his authority waned.

Every theory must look into morbid or rather pathological anatomy, to a greater or less extent, for a solid basis, for something like a panacea for the uncertainty of medicine: otherwise, it must continue to be a fruitful source of satire.

*New Orleans*, July, 1843.

